# Localization of Fréchet Frames and Expansion of Generalized Functions

**DOI:** 10.1007/s40840-020-01070-y

**Published:** 2021-02-23

**Authors:** Stevan Pilipović, Diana T. Stoeva

**Affiliations:** 1 Faculty of Sciences, Department of Mathematics and Informatics, University of Novi Sad, Novi Sad, Serbia; 2 Faculty of Mathematics, University of Vienna, Oskar-Morgenstern-Platz 1, Vienna, 1090 Austria; 3 Acoustics Research Institute, Austrian Academy of Sciences, Wohllebengasse 12-14, Vienna, 1040 Austria

**Keywords:** Localized frame, Banach frame, Fréchet frame, Tempered distributions, Ultradistributions, Frame expansions, 42C15, 46A13, 46B15, 46F05

## Abstract

Matrix-type operators with the off-diagonal decay of polynomial or sub-exponential types are revisited with weaker assumptions concerning row or column estimates, still giving the continuity results for the frame type operators. Such results are extended from Banach to Fréchet spaces. Moreover, the localization of Fréchet frames is used for the frame expansions of tempered distributions and a class of Beurling ultradistributions.

## Introduction, Motivation and Main Aims

Localized frames were introduced independently by Gröchenig [23] and Balan, Casazza, Heil, and Landau [2, 3]. The localization conditions in [23] are related to off-diagonal decay (of polynomial or exponential type) of the matrix determined by the inner products of the frame elements and the elements of a given Riesz basis. A localized frame in this sense leads to the same type of localization of the canonical dual frame as well as to the convergence of the frame expansions in all associated Banach spaces. We refer to [5, 13, 14, 20, 21], where various interesting properties and applications of localized frames were considered. The localization and self-localization, considered independently in [1–3], are directed to the over-completeness of frames and the relations between frame bounds and density with applications to Gabor frames. For the present paper, we have chosen to stick to the localization concept from [23], because the results obtained for a family of Banach spaces there can naturally be related to Fréchet frames (cf. [31–34]).

Our main aim in this paper is to present in Section 6 the frame expansions of tempered distributions and tempered ultradistributions of Beurling type by the use of localization. Matrix-type operators of Section 5 have an essential role in our investigations. The important novelty is the analysis related to sub-exponential off-diagonal decay without assumption of the exponential off-diagonal decay as it was considered in [23]. More precisely, in [23] the presumed exponential off-diagonal decay of matrices implies the analysis of sub-exponentially weighted spaces. Probably the most important impact in applications is related to the Hermite basis which is almost always used for the global expansion of $L^2$-functions or tempered generalized functions over ${{\mathbb {R}}}^n$. Our results by the use of localization, show that the same is true if one uses a kind of perturbation of Hermite functions through localization.

As particular results, not directly involved in the main ones, we extend in Sections 3 and 4 the continuity results on matrix-type operators acting on elements of a Banach or Fréchet spaces expanded by frames. We consider relaxed version of the classical off-diagonal decay conditions, assuming the column-decay and allowing row-increase in a matrix.

The paper is organized as follows. We recall in Section 2 the notation, basic definitions, and the needed known results. In Section 3, we consider matrices with column decay and possible row increase. For such type of matrices, we obtain in Section 4 continuity results for the frame related operators using less restrictive conditions in comparison with the localization conditions known in the literature. Sub-exponential localization is introduced and analyzed in Section 5. The use of Jaffard’s Theorem and [23, Theorems 11 and 13] is intrinsically connected with the sub-exponential localization. Section 6 is devoted to Fréchet frames and series expansions in certain classes of Fréchet spaces based on polynomial, exponential, and sub-exponential localization. In particular, we obtain frame expansions in the Schwartz space ${\mathcal {S}}$ of rapidly decreasing functions and its dual, the space of tempered distributions, as well as in the spaces $\Sigma ^\alpha$, $\alpha >1/2$, and their duals, spaces of tempered ultradistributions. In order to illustrate some results, we provide examples with the Hermite orthonormal basis $h_n, n\in {\mathbb {N}}$, and construct a Riesz basis which is polynomially and exponentially localized to $h_n, n\in {\mathbb {N}}$. Finally, in “Appendix”, we add some details in the proof of the Jaffard’s theorem.

## Notation, Definitions, and Preliminaries

Throughout the paper, $({\mathcal {H}}, \langle \cdot ,\cdot \rangle )$ denotes a separable Hilbert space and *G* (resp. *E*) denotes the sequence $(g_n)_{n=1}^\infty$ (resp. $(e_n)_{n=1}^\infty$) with elements from ${\mathcal {H}}$. Recall that *G* is called:

- *frame for *${\mathcal {H}}$ [15] if there exist positive constants *A* and *B* (called *frame bounds*) so that $A\Vert f\Vert ^2 \le \sum _{n=1}^\infty |\langle f,g_n\rangle |^2\le B\Vert f\Vert ^2$ for every $f\in {\mathcal {H}}$;
- *Riesz basis for *${\mathcal {H}}$ [4] if its elements are the images of the elements of an orthonormal basis under a bounded bijective operator on ${\mathcal {H}}$.

Recall (see, e.g., [12]), if *G* is a frame for ${\mathcal {H}}$, then there exists a frame $(f_n)_{n=1}^\infty$ for ${\mathcal {H}}$ so that

$$\begin{aligned} f=\sum _{n=1}^\infty \langle f,f_n\rangle g_n=\sum _{n=1}^\infty \langle f,g_n\rangle f_n, f\in {\mathcal {H}}. \end{aligned}$$

Such $(f_n)_{n=1}^\infty$ is called a *dual frame of *$(g_n)_{n=1}^\infty$. Furthermore, the *analysis operator* $U_G$, given by $U_Gf=(\langle f, g_n\rangle )_{n=1}^\infty$, is bounded from ${\mathcal {H}}$ into $\ell ^2$; the *synthesis operator* $T_G$, given by $T_Gf=\sum _{n=1}^\infty c_n g_n$, is bounded from $\ell ^2$ into ${\mathcal {H}}$; the *frame operator* $S_G:=T_G U_G$ is a bounded bijection of ${\mathcal {H}}$ onto ${\mathcal {H}}$ with unconditional convergence of the series $S_G f =\sum _{n=1}^\infty \langle f, g_n\rangle g_n$. The sequence $(S_G^{-1}g_n)_{n=1}^\infty$ is a dual frame of $(g_n)_{n=1}^\infty$, called the *canonical dual of* $(g_n)_{n=1}^\infty$, and it will be denoted by $(\widetilde{g_n})_{n=1}^\infty$ or $\widetilde{G}$. When *G* is a Riesz basis of ${\mathcal {H}}$ (and thus a frame for ${\mathcal {H}}$), then only $\widetilde{G}$ is a dual frame of *G*, it is the unique biorthogonal sequence to *G*, and it is also a Riesz basis for ${\mathcal {H}}$. A frame *G* which is not a Riesz basis has other dual frames in addition to the canonical dual and in that case we use notation $G^d$ or $(g_n^d)_{n=1}^\infty$ for a dual frame of *G*.

Next, $(X, \Vert \cdot \Vert )$ denotes a Banach space and $( \Theta , {\Vert |{\cdot }\Vert |})$ denotes a Banach sequence space; $\Theta$ is called a *BK*-*space* if the coordinate functionals are continuous. If the canonical vectors form a Schauder basis for $\Theta$, then $\Theta$ is called a *CB*-*space*. A *CB*-space is clearly a *BK*-space.

Given a *BK*-space $\Theta$ and a frame *G* for ${\mathcal {H}}$ with a dual frame $G^d=(g_n^d)_{n=1}^\infty$, one associates with $\Theta$ the Banach space

$$\begin{aligned} {\mathcal {H}}^{\Theta }_{G, G^d} := \left\{ f\in {\mathcal {H}}\ : \ (\langle f, g^d_n\rangle )_{n=1}^\infty \in \Theta , \, \Vert f\Vert _{{\mathcal {H}}^{\Theta }_{G, G^d}}:={\Vert |{ (\langle f, g_n^d\rangle )_{n=1}^\infty }\Vert |}_\Theta \right\} . \end{aligned}$$

When *G* is a Riesz basis for ${\mathcal {H}}$, then we use notation ${\mathcal {H}}^\Theta _G$ for ${\mathcal {H}}^{\Theta }_{G, \widetilde{G}}$.

### Localization of Frames

In this paper, we consider polynomially and exponentially localized frames in the way defined in [23], and furthermore, sub-exponential localization. Let *G* be a Riesz basis for the Hilbert space ${\mathcal {H}}$. A frame *E* for ${\mathcal {H}}$ is called:

- *polynomially localized with respect to G* *with decay *$\gamma >0$ (in short, $\gamma$*-localized wrt *$(g_n)_{n=1}^\infty$) if there is a constant $C_\gamma >0$ so that
  $$\begin{aligned}  \max \{ |\langle e_m, g_n\rangle |, |\langle e_m, \widetilde{g_n}\rangle |\} \le C_\gamma (1+|m-n|)^{-\gamma }, \ m,n\in {\mathbb {N}}; \end{aligned}$$
- *exponentially localized with respect to G* if for some $\gamma >0$ there is a constant $C_\gamma >0$ so that
  $$\begin{aligned} \max \{ |\langle e_m, g_n\rangle |, |\langle e_m, \widetilde{g_n}\rangle |\} \le C_\gamma \mathrm {e}^{-\gamma |m-n|}, \ m,n\in {\mathbb {N}}. \end{aligned}$$
- $\beta$*-sub-exponentially localized with respect to G* (for $\beta \in (0,1)$) if for some $\gamma >0$ there is $C_\gamma >0$ so that
  $$\begin{aligned} \max \{ |\langle e_m, g_n\rangle |, |\langle e_m, \widetilde{g_n}\rangle |\} \le C_\gamma \mathrm {e}^{- \gamma |m-n|^\beta }, \ m,n\in {\mathbb {N}}. \end{aligned}$$

### Fréchet Frames

We consider Fréchet spaces which are projective limits of Banach spaces as follows. Let $\{Y_k, | \cdot |_k\}_{k\in {\mathbb {N}}_0}$ be a sequence of separable Banach spaces such that

1 $$\begin{aligned}&\{{\mathbf{0}}\} \ne \cap _{k\in {\mathbb {N}}_0} Y_k \subseteq \ldots \subseteq Y_2 \subseteq Y_1 \subseteq Y_0, \; \ |\cdot |_0\le | \cdot |_1\le | \cdot |_2\le \ldots \end{aligned}$$

2 $$\begin{aligned}&Y_F :=\cap _{k\in {\mathbb {N}}_0} Y_k \;\; \text{ is } \text{ dense } \text{ in } Y_k, \;\; k\in {\mathbb {N}}_0\; ({\mathbb {N}}_0={\mathbb {N}}\cup \{0\}). \end{aligned}$$

Under the conditions (1)–(2), $Y_F$ is a Fréchet space and $Y_F^*$ is the inductive limit of the spaces $Y_k^*$, $k\in {\mathbb {N}}$. We will use such type of sequences in two cases: 1. $Y_k=X_k$ with norm $\Vert \cdot \Vert _k, k\in {\mathbb {N}}_0;$ 2. $Y_k=\Theta _k$ with norm ${\Vert |{\cdot }\Vert |}_k, k\in {\mathbb {N}}_0$.

Let $\{\Theta _k, {\Vert |{\cdot }\Vert |}_k\}_{k\in {\mathbb {N}}_0}$ be a sequence of *CB*-spaces satisfying (1). Then (2) holds, because every sequence $(c_n)_{n=1}^\infty \in \Theta _F$ can be written as $\sum _{n=1}^\infty c_n \delta _n$ with the convergence in $\Theta _F$, where $\delta _n$ denotes the *n*-th canonical vector, $n\in {\mathbb {N}}$. Furthermore, $\Theta _F^*$ can be identified with the sequence space $\Theta _F^{\circledast }:=\{(U\delta _n)_{n=1}^\infty \ : \ U\in \Theta _F^*\}$ with convergence naturally defined in correspondence with the convergence in $\Theta _F^*$.

We use the term *operator* for a linear mapping, and by *invertible operator on X*, we mean a bounded bijective operator on *X*. Given sequences of Banach spaces, $\{X_k\}_{k\in {\mathbb {N}}_0}$ and $\{\Theta _k\}_{k\in {\mathbb {N}}_0}$, which satisfy (1)-(2), an operator $T: \Theta _F \rightarrow X_F$ is called *F-bounded* if for every $k\in {\mathbb {N}}_0$, there exists a constant $C_k>0$ such that $\Vert T(c_n)_{n=1}^\infty \Vert _k\le C_k {\Vert |{(c_n)_{n=1}^\infty }\Vert |}_k$ for all $(c_n)_{n=1}^\infty \in \Theta _F$.

#### Definition 2.1

[34] Let $\{X_k, \Vert \cdot \Vert _k\}_{k\in {\mathbb {N}}_0}$ be a sequence of Banach spaces satisfying (1)-(2) and let $\{\Theta _k, {\Vert |{\cdot }\Vert |}_k\}_{k\in {\mathbb {N}}_0}$ be a sequence of *BK*-spaces satisfying (1)-(2). A sequence $(\phi _n)_{n=1}^\infty$ with elements from ${X_F^*}$ is called a *General Fréchet frame* (in short, *General F-frame*) for $X_F$ with respect to $\Theta _F$ if there exist sequences $\{\widetilde{s}_k\}_{k\in {\mathbb {N}}_0}\subseteq {\mathbb {N}}_0$, $\{s_k\}_{k\in {\mathbb {N}}_0}\subseteq {\mathbb {N}}_0$, which increase to $\infty$ with the property $s_k\le \widetilde{s}_k$, $k\in {\mathbb {N}}_0$, and there exist constants $0<A_k\le B_k<\infty$, $k\in {\mathbb {N}}_0$, satisfying

3 $$\begin{aligned}&(\phi _n(f))_{n=1}^\infty \in \Theta _F, \ f\in X_F, \end{aligned}$$

4 $$\begin{aligned}&A_k \Vert f\Vert _{s_k}\le {\Vert |{(\phi _n(f))_{n=1}^\infty }\Vert |}_k\le B_k\Vert f\Vert _{\widetilde{s}_k}, \ f\in X_F, k\in {\mathbb {N}}_0, \end{aligned}$$

and there exists a continuous operator $V:\Theta _F\rightarrow X_F$ so that $V((\phi _n(f))_{n=1}^\infty )=f$ for every $f\in X_F$.

When $s_k=\widetilde{s}_k=k$, $k\in {\mathbb {N}}_0$, and the continuity of *V* is replaced by the stronger condition of *F*-boundedness of *V*, then the above definition reduces to the definition of a *Fréchet frame* (in short, *F-frame*) for $X_F$ with respect to $\Theta _F$ introduced in [32]. Although we will use in the sequel this simplified definition, Definition 2.1 is the most general one, interesting in itself, and can be considered as a non-trivial generalization of Banach frames.

In the particular case when $X_k=X$, and $\Theta _k=\Theta$, $k\in {\mathbb {N}}_0$, a Fréchet frame for $X_F$ with respect to $\Theta _F$ becomes a *Banach frame for X* *with respect to* $\Theta$ as introduced in [22].

For another approach to frames in Fréchet spaces, we refer to [6]. For more on frames for Banach spaces, see, e.g., [8, 9, 37] and the references therein.

### Sequence and Function Spaces

Recall that a positive continuous function $\mu$ on ${\mathbb {R}}$ is called: a *k-moderate weight* if $k\ge 0$ and there exists a constant $C>0$ so that $\mu (t+x)\le C (1+|t|)^k \mu (x), \ t,x\in {\mathbb {R}};$ a $\beta$*-sub-exponential* (resp. *exponential*) weight, if $\beta \in (0,1)$ (resp. $\beta =1$) and there exist constants $C>0,\gamma >0$, so that $\mu (t+x)\le C e^{\gamma |t|^\beta }\mu (x), \ t,x\in {\mathbb {R}}.$ If $\beta$ is clear from the context, we will write just *sub-exponential weight*. Let $\mu$ be a *k*-moderate, sub-exponential, or exponential weight so that $\mu (n)\ge 1$ for every $n\in {\mathbb {N}}$, and $p\in [1,\infty )$. Then the Banach space

$$\begin{aligned} \ell ^p_{\mu }:=\left\{ (a_n)_{n=1}^\infty \ : {\Vert |{(a_n)_n}\Vert |}_{p,\mu } := \left( \sum _{n=1}^\infty |a_n|^p \mu (n)^{p}\right) ^{1/p}<\infty \right\} \end{aligned}$$

is a *CB*-space. We refer, for example, to [28, Ch. 27] for the so-called Köthe sequence spaces.

We will need the following lemma, which can be easily proved by the use of [32, Theor. 4.2].

#### Lemma 2.2

Let *G* be a frame for ${\mathcal {H}}$ and let $G^d=(g^d_n)_{n=1}^\infty$ be a dual frame of *G*. Let $\mu _k$ be *k*-moderate (resp. sub-exponential or exponential) weights, $k\in {\mathbb {N}}_0,$ so that

5 $$\begin{aligned} 1=\mu _0(x)\le \mu _1(x) \le \mu _2(x) \le ..., \ \text{ for } \text{ every } x\in {\mathbb {R}}. \end{aligned}$$

Then the spaces $\Theta _k:=\ell ^2_{\mu _k}$, $k\in {\mathbb {N}}_0$, satisfy (1)–(2). Denote $M:= \{ (\langle f,g^d_n\rangle )_{n=1}^\infty \ : \ f\in {\mathcal {H}}\}$. The assumption that $M \cap \Theta _F$ is dense in $M \cap \Theta _k\ne \{0\}$ with respect to the ${\Vert |{\cdot }\Vert |}_k$-norm for every $k\in {\mathbb {N}}$, leads to the conclusion that the spaces $X_k:={\mathcal {H}}_{G,G^d}^{\Theta _k}$, $k\in {\mathbb {N}}$, satisfy (1)–(2).

If *G* is a Riesz basis for ${\mathcal {H}}$, then the density assumption of $M \cap \Theta _F$ in $M \cap \Theta _k\ne \{0\}$, $k\in {\mathbb {N}}$, is fulfilled and in addition one has that $g_n\in X_F$ for every $n\in {\mathbb {N}}$.

Throughout the paper, we also consider specific weights, relevant to the function spaces of interest and the corresponding sequence spaces. Let $k\in {\mathbb {N}}_0$ and $\mu _k(x)=(1+|x|)^k$ (resp. $\mu _k(x)=e^{k|x|^\beta }, \beta \in (0,1]$), $x\in {\mathbb {R}}$. Then, with $\Theta _k:=\ell ^p_{\mu _k}$, $k\in {\mathbb {N}}_0$, the projective limit $\cap _{k\in {\mathbb {N}}_0} \Theta _k$ is the space $\mathbf{s}$ of rapidly (resp. ${\mathfrak {s}}^{\beta }$ of sub-exponentially when $\beta <1$ and exponentially when $\beta =1$) decreasing sequences determined by $\{ (a_n)_{n=1}^\infty \in {\mathbb {C}}^{\mathbb {N}}\ : \ (\sum _{n=1}^\infty |a_n\mu _k(n))|^p)^{1/p} <\infty , \ \forall k\in {\mathbb {N}}_0\}$, which is the same set for any $p\in [1,\infty )$. The space $\mathbf{s}$ (resp. ${\mathfrak {s}}^\beta$) can also be derived as the projective limit of the Banach spaces $\mathbf{s}_k$ (resp. ${\mathfrak {s}}^\beta _k$) defined as $\{(a_n)_{n=1}^\infty \in {\mathbb {C}}^{\mathbb {N}}\ : \ {\Vert |{(a_n)_{n=1}^\infty }\Vert |}_{\sup ,k}:= \sup _{n\in {\mathbb {N}}} |a_n|n^k <\infty \}$, (resp. ${\Vert |{\cdot }\Vert |}_{\sup ,k}^\beta := \sup _{n\in {\mathbb {N}}} |a_n| e^{kn^{\beta }} <\infty \}$), $k\in {\mathbb {N}}_0$; note that here instead of $k\in \{0,1,2,3,\ldots \}$ one can also use any strictly increasing sequence of nonnegative numbers $k\in \{0, q_1, q_2, q_3, \ldots \}$.

Recall that the well-known Schwartz space $\mathcal {S}$ is the intersection of Banach spaces

$$\begin{aligned} {{\mathcal {S}}}_k ({\mathbb {R}}) := \left\{ f\in L^2({\mathbb {R}}):||f||_k=\sum _{m=0}^k||(1+|\cdot |^2)^{k/2}f^{(m)}||_{L^2({\mathbb {R}})} \right\} , k\in {\mathbb {N}}. \end{aligned}$$

The dual ${{\mathcal {S}}}' ({\mathbb {R}})$ is the space of tempered distributions.

The space of sub-exponentially decreasing functions of order $1/\alpha$, $\alpha >1/2,$ is $\Sigma ^\alpha :=X_F= \cap _{k\in {\mathbb {N}}_0}\Sigma ^{k,\alpha }$ where $\Sigma ^{k,\alpha }$ are Banach spaces of $L^2-$ functions with finite norms

$$\begin{aligned} ||f||_k^\alpha =\sup _{n\in {\mathbb {N}}_0} \left\| \frac{k^{n}e^{k|x|^{1/\alpha }}|f^{(n)}(x)|}{ n!^\alpha }\right\| _{L^2({\mathbb {R}})}<\infty , k\in {\mathbb {N}}. \end{aligned}$$

Its dual $({\Sigma ^\alpha }({\mathbb {R}}))'$ is the space of Beurling tempered ultradistributions, cf. [19, 30].

#### Remark 2.3

The case $\alpha =1/2$ leads to the trivial space $\Sigma ^{1/2}=\{0\}.$ There is another way in considering the test space which corresponds to that limiting Beurling case $\alpha =1/2$ and can be considered also for $\alpha <1/2$ (cf. [10, 11, 19, 29]). We will not treat these cases in the current paper.

In the sequel, $(h_n)_{n=1}^\infty$ is the Hermite orthonormal basis of $L^2({\mathbb {R}})$ re-indexed from 1 to $\infty$ instead of from 0 to $\infty$. Recall that $h_n\in \Sigma ^\alpha$, $\alpha >1/2$, $n\in {\mathbb {N}}$. Moreover, we know [36]:

- If $f\in \mathcal {S}$, then $(\langle f, h_n\rangle )_{n=1}^\infty \in \mathbf{s}$; conversely, if $(a_n)_{n=1}^\infty \in \mathbf{s}$, then $\sum _{n=1}^\infty a_n h_n$ converges in $\mathcal {S}$ to some *f* with $(\langle f, h_n\rangle )_{n=1}^\infty =(a_n)_{n=1}^\infty$.
- If $F\in \mathcal {S}'$, then $(b_n)_{n=1}^\infty :=(F(h_n))_{n=1}^\infty \in \mathbf{s}'$ and $F(f)=\sum _{n=1}^\infty \langle f, h_n\rangle b_n$, $f\in \mathcal {S}$; conversely, if $(b_n)_{n=1}^\infty \in \mathbf{s}'$, then the mapping $F: f\rightarrow \sum _{n=1}^\infty \langle f, h_n\rangle b_n$ is well defined on $\mathcal {S}$, it determines *F* as an element of $\mathcal {S}'$ and $(F(h_n))_{n=1}^\infty =(b_n)_{n=1}^\infty$.

The above two statements also hold when $\mathcal {S}$, $\mathcal {S}'$, $\mathbf{s}$, and $\mathbf{s}'$ are replaced by $\Sigma ^\alpha$, $(\Sigma ^\alpha )'$, ${\mathfrak {s}}^{1/(2\alpha )}$, and $({\mathfrak {s}}^{1/(2\alpha )})'$ with $\alpha >1/2$, respectively [19, 29, 30].

We can consider $\mathcal {S}$ and $\Sigma ^\alpha$ as the projective limit of Hilbert spaces ${\mathcal {H}}^k, k\in {\mathbb {N}}_0,$ with elements $f=\sum _n a_nh_n,$ in the first case with norms

$$\begin{aligned} \Vert f\Vert _{{\mathcal {H}}^k}:={\Vert |{(a_nn^k)_n}\Vert |}_{\ell ^2}<\infty \}, k\in {\mathbb {N}}_0, \end{aligned}$$

and in the second case with norms

$$\begin{aligned} \Vert f\Vert _{{\mathcal {H}}^k}:={\Vert |{(a_ne^{kn^{1/(2\alpha )}})_n}\Vert |}_{\ell ^2}<\infty \}, k\in {\mathbb {N}}_0. \end{aligned}$$

Thus, $(h_n)_n$ is an *F*-frame for ${\mathcal {S}}({\mathbb {R}})$ with respect to $\mathbf{s}$ as well as an *F*-frame for $\Sigma ^\alpha$ with respect to ${\mathfrak {s}}^{1/2\alpha }$, $\alpha >1/2$, (*F*- boundedness is trivial).

## Matrix-Type Operators

Papers [14, 21, 23] concern matrices with off-diagonal decay of the form: for some $\gamma >0$ there is $C_\gamma >0$ such that

6 $$\begin{aligned} |A_{m,n}|\le \frac{C_\gamma }{(1+|m-n|)^\gamma } \ \ \ (\hbox {resp. } |A_{m,n}|\le C_\gamma \mathrm {e}^{-\gamma |m-n|}),\ \forall n,m\in {\mathbb {N}}. \end{aligned}$$

As we noted in the introduction, matrix operators in this section and the next one are not essentially related to Sections 5 and 6. But they significantly illuminate such operators in our main results. Moreover, we refer in Section 4 to results of Section 3 and in Remark 6.4 we refer to Section 4.

We will consider matrices with more general off-diagonal type of decay (see $$(***)$$ below which is weaker condition compare to the polynomial type condition in (6)). Moreover, we consider matrices which have column decrease but allow row increase (see Propositions 3.2 and 3.6) allowing sub-exponential type conditions as well. For such more general matrices, we generalize some results from [23] with respect to certain Banach spaces and, furthermore, proceed to the Fréchet case.

In the sequel, for a given matrix $(A_{mn})_{m,n\in {\mathbb {N}}}$, the letter $\mathcal {A}$ will denote the mapping $(c_n)_{n=1}^\infty \rightarrow (a_m)_{m=1}^\infty$ determined by $a_m=\sum _{n=1}^\infty A_{m,n} c_n$ (assuming convergence), $m\in {\mathbb {N}}$; conversely, for a given mapping $\mathcal {A}$ determined on a sequence space containing the canonical vectors $\delta _n$, $n\in {\mathbb {N}}$, the corresponding matrix $(A_{mn})_{m,n\in {\mathbb {N}}}$ is given by $A_{m,n}=\langle \mathcal {A}\delta _n, \delta _m\rangle$. We will sometimes use $\mathcal {A}$ with the meaning of $(A_{mn})_{m,n\in {\mathbb {N}}}$ and vice-verse.

### Polynomial-Type Conditions

Let us begin with some comparison of polynomial type of off-diagonal decay:

#### Lemma 3.1

Let $\gamma >0$. Consider the following conditions:

$$\begin{aligned}&(*) \quad |A_{m,n}|\le C \left\{ \begin{array}{ll} \frac{(1+ m)^\gamma }{(1+n)^{2\gamma }}, &{} n\ge m, \\ \frac{(1+ n)^\gamma }{(1+m)^{2\gamma }}, &{} n\le m, \text{ for } \text{ some } C>0. \end{array} \right. \\&(**) \quad |A_{m,n}| \le C(1+|n-m|)^{-\gamma }, \text{ for } \text{ some } C>0. \\&(***) \quad |A_{m,n}|\le C\left\{ \begin{array}{ll} \frac{m^\gamma }{n^\gamma }, &{} n\ge m, \\ \frac{n^\gamma }{m^\gamma }, &{} n\le m, \text{ for } \text{ some } C>0. \end{array} \right. \end{aligned}$$

Then, the implications $$(*)\Rightarrow (**)\Rightarrow (***)$$ hold. The converse implications are not valid.

#### Proof

Implications $$(*)\Rightarrow (**)\Rightarrow (***)$$ follow from the inequalities $$\frac{(1+ min(m,n))^\gamma }{(1+max(m,n))^{2\gamma }}\le (1+|n-m|)^{-\gamma } \le \frac{(min(m,n))^\gamma }{(max(m,n))^\gamma }, n,m\in {\mathbb {N}},$$ which are easy to be verified. To show that $$ (***)$$ does not imply $$(**)$$ even up to a multiplication with a constant, take a matrix $$A_{m,n}$$ which satisfies $$|A_{m,n}|= \frac{C n^\gamma }{m^\gamma },\ n\le m$$, for some $$\gamma >0$$ and some positive constant *C*,  and assume that there exist $$\gamma _1(\gamma )\in {\mathbb {N}}$$ and a positive constant *K* so that for $$m\ge n$$ one has $$\frac{C n^\gamma }{m^\gamma } \le \frac{K}{ (1+m-n)^{\gamma _1}}$$; then taking $$m=2n$$, one obtains $$0<C \cdot 2^{-\gamma } \le \frac{K}{(1+n)^{\gamma _1}}\rightarrow 0 \text{ as } n\rightarrow \infty $$, which leads to a contradiction. In a similar spirit, one can show that $$ (**)$$ does not imply $$(*)$$. $$\square $$

Below we show that the relaxed polynomial-type conditions, as well as conditions allowing row-increase, still lead to continuous operators.

#### Proposition 3.2

Assume that the matrix $(A_{mn})_{m,n\in {\mathbb {N}}}$ satisfies the condition

$$\begin{aligned} |A_{m,n}|\le \left\{ \begin{array}{ll} C_0\, n^{\gamma _0}, &{} n>m, \\ C_1 \,n^{\gamma _1} m^{-\gamma _1}, &{} n\le m, \end{array} \right. \end{aligned}$$

for some $\gamma _0\ge 0, \gamma _1>0, C_0>0, C_1>0$. Then $\mathcal {A}$ is a continuous operator from $\mathbf{s}_{\gamma _1+\gamma _0+1+\varepsilon }$ into $\mathbf{s}_{\gamma _1}$ for any $\varepsilon \in (0,1]$.

#### Proof

Let $\varepsilon \in (0,1]$ and let $(c_n)_{n=1}^\infty \in \mathbf{s}_{\gamma _0+\gamma _1+1+\varepsilon }$. For every $n>m$,

$$\begin{aligned} |A_{m,n} c_n|\le & {} C_0 |c_n| n^{\gamma _0} \le C_0 \left( \sup _j (|c_j| j^{\gamma _0+\gamma _1 +1+\varepsilon })\right) \frac{1}{n^{\gamma _1+1+\varepsilon }}. \end{aligned}$$

Next,

$$\begin{aligned} |\sum _{n=1}^m A_{m,n} c_n|\le & {} C_1 m^{-\gamma _1} \sum _{n=1}^m |c_n| n^{\gamma _1}\\\le & {} C_1 m^{-\gamma _1} {\Vert |{(c_n)_{n=1}^\infty }\Vert |}_{\sup ,\gamma _0+\gamma _1+1+\varepsilon } \sum _{n=1}^m \frac{1}{n^{\gamma _0+1+\varepsilon }}. \end{aligned}$$

Therefore,

$$\begin{aligned} |a_m|\le & {} |\sum _{n=1}^m A_{m,n} c_n| + |\sum _{n=m+1}^\infty A_{m,n} c_n| \\\le & {} C_1 m^{-\gamma _1} {\Vert |{(c_n)_{n=1}^\infty }\Vert |}_{\sup ,\gamma _0+\gamma _1+1+\varepsilon } \sum _{n=1}^\infty \frac{1}{n^{\gamma _0+1+\varepsilon }} \\&+ C_0 {\Vert |{(c_n)_{n=1}^\infty }\Vert |}_{\sup ,\gamma _0+\gamma _1+1+\varepsilon } \sum _{n=m+1}^\infty \frac{1}{ n^{\gamma _1+1+\varepsilon }}. \end{aligned}$$

Since $\sum _{n=m+1}^\infty \frac{1}{ n^{\gamma _1+1+\varepsilon }}\le m^{-\gamma _1} \sum _{n=m+1}^\infty \frac{1}{ n^{1+\varepsilon }},$ the assertion follows. $\square$

A direct consequence of Proposition 3.2 is:

#### Corollary 3.3

Assume that the matrix $(A_{mn})_{m,n\in {\mathbb {N}}}$ satisfies: there exist $\gamma _0\ge 0$ and $C_0>0$, and for every $\gamma >0$ there is $C_\gamma >0$ so that

$$\begin{aligned} |A_{m,n}|\le \left\{ \begin{array}{ll} C_0 n^{\gamma _0}, &{} n>m \\ C_\gamma n^\gamma m^{-\gamma }, &{} n\le m. \end{array} \right. \end{aligned}$$

Then $\mathcal {A}$ is a continuous operator from $\mathbf{s}$ into $\mathbf{s}$.

In order to determine $\mathcal {A}$ as a mapping from a space $\mathbf{s}_{\gamma _1}$ into the same space, we have to change the decay condition.

#### Proposition 3.4

Let $$(A_{mn})_{m,n\in {\mathbb {N}}}$$ satisfy:

$$\begin{aligned} (\exists \varepsilon>0, \gamma _1\in {\mathbb {N}}) (\exists C_0>0, C_1>0) \text{ such } \text{ that } \end{aligned}$$

7 $$\begin{aligned} |A_{m,n}|\le \left\{ \begin{array}{ll} C_0n^{-1-\varepsilon } , &{} n> m, \\ C_1 n^{\gamma _1} m^{-\gamma _1-1-\varepsilon }, &{} n\le m. \end{array} \right. \end{aligned}$$

Then $\mathcal {A}$ is a continuous operator from $\mathbf{s}_{\gamma _1}$ into $\mathbf{s}_{\gamma _1}$.

#### Remark 3.5

For the same conclusion as above, one has in [23] another condition non-comparable to (7):

8 $$\begin{aligned} |A_{m,n}|\le C(1+|n-m|)^{-\gamma _1-1-\varepsilon }. \end{aligned}$$

### Sub-exponential- and Exponential-Type Conditions

Up to the end of the paper $\beta$ will be a fixed number of the interval (0, 1];  $\beta =1$ is related to the exponential growth order while $\beta \in (0,1)$ corresponds to the pure sub-exponential growth order.

#### Proposition 3.6

Assume that the matrix $(A_{mn})_{m,n\in {\mathbb {N}}}$ satisfies the condition: There exist positive constants $C_0, C_1$ and $\gamma _0\ge 0$, $\gamma _1>0$, so that

9 $$\begin{aligned} |A_{m,n}|\le \left\{ \begin{array}{ll} C_0 \mathrm {e}^{\gamma _0n^{\beta }}, &{} n>m, \\ C_1 \mathrm {e}^{-\gamma _1 (m^\beta -n^\beta )}, &{} n\le m. \end{array} \right. \end{aligned}$$

Then $\mathcal {A}$ is a continuous operator from ${\mathfrak {s}}^\beta _{\gamma _1+\gamma _0+\varepsilon }$ into ${\mathfrak {s}}^\beta _{\gamma _1}$ for any $\varepsilon \in (0,1)$.

#### Proof

Let $\varepsilon \in (0,1)$ and let $(c_n)_{n=1}^\infty \in {\mathfrak {s}}^\beta _{\gamma _1+\gamma _0+\varepsilon }$. Then for $n>m$,

$$\begin{aligned} |A_{m,n}c_{n}|\le & {} C_0 |c_{n}| \mathrm {e}^{\gamma _0 n^\beta } \le C_0 (\sup _j |c_j| \mathrm {e}^{(\gamma _1+\gamma _0+\varepsilon ) j^\beta }) \mathrm {e}^{-(\gamma _1+\varepsilon ) n^\beta }. \end{aligned}$$

Further on,

$$\begin{aligned} |\sum _{n=1}^m A_{m,n} c_n|\le & {} C_1 \sum _{n=1}^m |c_n| \mathrm {e}^{\gamma _1(n^\beta -m^\beta )} \\\le & {} C_1 \mathrm {e}^{-\gamma _1 m^\beta } (\sup _{j\in {\mathbb {N}}} |c_j| \mathrm {e}^{(\gamma _1+\gamma _0+\varepsilon ) j^\beta }) \sum _{n=1}^m \mathrm {e}^{-(\gamma _0 +\varepsilon )n^\beta }. \end{aligned}$$

Therefore,

$$\begin{aligned} |a_m|\le & {} \mathrm {e}^{-\gamma _1 m^\beta } \left( C_1 \sum _{n=1}^\infty \mathrm {e}^{-(\gamma _0+\varepsilon ) n^\beta } + C_0 \sum _{n=1}^\infty \mathrm {e}^{-\varepsilon n^\beta }\right) {\Vert |{(c_n)_n}\Vert |}_{\sup ,\gamma _1+\gamma _0+\varepsilon }^\beta . \end{aligned}$$

This completes the proof. $\square$

#### Remark 3.7

Since $e^{-\gamma (m-n)^\beta } \le e^{-\gamma (m^\beta -n^\beta )}$ for $n\le m$ ($\beta \in (0,1], \gamma \in (0,\infty )$), in (9) we consider $e^{-\gamma (m^\beta -n^\beta )}$ instead of $e^{-\gamma {(m-n)^\beta }}.$

As a consequence of Proposition 3.6, we have:

#### Corollary 3.8

Assume that the matrix $(A_{mn})_{m,n\in {\mathbb {N}}}$ satisfies the condition: There exist constants $C_0>0$ and $\gamma _0\ge 0$, and for every $\gamma >0$, there is a positive constant $C_\gamma$ so that

$$\begin{aligned} |A_{m,n}|\le \left\{ \begin{array}{ll} C_0 {e}^{\gamma _0n^{\beta }}, &{} n>m, \\ C_\gamma e^{\gamma (n^\beta -m^\beta )}, &{} n\le m. \end{array} \right. \end{aligned}$$

Then $\mathcal {A}$ is a continuous operator from ${\mathfrak {s}}^\beta$ into ${\mathfrak {s}}^\beta$.

#### Proposition 3.9

Let $(A_{mn})_{m,n\in {\mathbb {N}}}$ satisfy the condition: There exist positive constants $\varepsilon , \gamma _1, C_0, C_1$, so that

$$\begin{aligned} |A_{m,n}|\le \left\{ \begin{array}{ll} C_0e^{-\varepsilon n^\beta } , &{} n> m, \\ C_1e^{\gamma _1 n^{\beta }} e^{-(\gamma _1 +\varepsilon )m^{\beta }}, &{} n\le m. \end{array} \right. \end{aligned}$$

Then $\mathcal {A}$ is a continuous operator from ${\mathfrak {s}}^\beta _{\gamma _1}$ into ${\mathfrak {s}}^\beta _{\gamma _1}$.

#### Remark 3.10

One can simply show that the assumption $|A_{m,n}|\le C e^{-\gamma |m-n|^\beta }, m,n\in {\mathbb {N}}$, leads to similar continuity results. We will consider this condition later in relation to the invertibility of such matrices and the Jaffard theorem.

## Continuity of the Frame-Related Operators Under Relaxed “Decay” Conditions

We now determine weaker localization conditions which are still sufficient to imply continuity of the frame-related operators.

### Proposition 4.1

Let *G* be a frame for ${\mathcal {H}}$, $G^d$ be a dual frame of *G*, and $\mu _k(x) = (1+|x|)^k$, $k\in {\mathbb {N}}_0$. Under the notations in Lemma 2.2, assume that $M \cap \Theta _F$ is dense in $M \cap \Theta _k\ne \{0\}$ with respect to the ${\Vert |{\cdot }\Vert |}_k$-norm for every $k\in {\mathbb {N}}$ and let $E=(e_n)_{n=1}^\infty$ be a sequence with elements from $X_F$ which is a frame for ${\mathcal {H}}$. Then the following statements hold.

(i) Assume that there exist $s_0\in {\mathbb {N}}$, $C>0$ and for every $k\in {\mathbb {N}}$ there exists $C_k>0$ such that
  $$\begin{aligned} |\langle e_m, g_n\rangle | \le \left\{ \begin{array}{ll} Cn^{s_0}, &{} n>m, \\ C_k n^k m^{-k}, &{} n\le m. \end{array} \right. \end{aligned}$$
  Then the analysis operator $f\rightarrow U_Ef =(\langle f,e_m\rangle )_{m=1}^\infty$ is continuous from $X_F$ into $\mathbf{s}$.
(ii) Assume that there exist $\widetilde{s}_0\in {\mathbb {N}}_0$, $\widetilde{C}>0$ and for every $k\in {\mathbb {N}}$ there exists $\widetilde{C}_k>0$ such that
  $$\begin{aligned} |\langle e_m, g^d_n\rangle |\le \left\{ \begin{array}{ll} \widetilde{C} m^{\widetilde{s}_0}, &{} m>n, \\ \widetilde{C}_k m^k n^{-k}, &{} m\le n. \end{array} \right. \end{aligned}$$
  Then the synthesis operator $(c_n)_n\rightarrow T_E(c_n) =\sum c_n e_n$ is continuous from $\mathbf{s}$ into $X_F$.
(iii) Under the assumptions of (i) and (ii), the frame operator $T_E U_E$ is continuous from $X_F$ into $X_F$.

### Proof

Note that under the given assumptions, $\Theta _F$ is the space $\mathbf{s}$.

(i) Let $A_{m,n}=\langle g_n, e_m\rangle$, $m,n\in {\mathbb {N}}$, and $\mathcal {A}$ be the corresponding operator for the matrix *A*. Let $f\in X_F$. Then $(\langle f, g^d_n\rangle )_{n=1}^\infty \in \mathbf{s}$ and
  $$\begin{aligned} \mathcal {A} (\langle f, g^d_n\rangle )_{n=1}^\infty = \left( \sum _{n=1}^\infty \langle g_n, e_m\rangle \langle f, g^d_n\rangle \right) _{m=1}^\infty =(\langle f,e_m\rangle )_{m=1}^\infty . \end{aligned}$$
  By Corollary 3.3, it follows that $(\langle f,e_m\rangle )_{n=1}^\infty \in \mathbf{s}$. Furthermore, by Proposition 3.2, for every $k\in {\mathbb {N}}$, there is a constant $K_{s_0,k,C,C_k}$ so that
  $$\begin{aligned} {\Vert |{(\langle f, e_m\rangle )_m}\Vert |}_{\sup ,k}= & {} {\Vert |{\mathcal {A}(\langle f, g^d_n\rangle )_n}\Vert |}_{\sup ,k}\le K_{s_0,k,C,C_k} {\Vert |{(\langle f, g^d_n\rangle )_n}\Vert |}_{\sup ,s_0 + k+2}\\\le & {} K_{s_0,k,C,C_k} \, {\Vert |{(\langle f, g^d_n\rangle )_n}\Vert |}_{\Theta _{s_0+k+2}} =K_{s_0,k,C,C_k} \, \Vert f\Vert _{s_0 + k+2}. \end{aligned}$$
  Therefore, the analysis operator $U_E$ is continuous from $X_F$ into $\mathbf{s}$.
(ii) Let $(c_n)\in \mathbf{s}$. First we show that $\sum _{n=1}^\infty c_n e_n$ converges in $X_F$ and then the continuity of $T_E$. Since $(c_n)_{n=1}^\infty \in \ell ^2$, we have $x=\sum _n c_n e_n\in {\mathcal {H}}$. Denote $A_{m,n}=\langle e_n, g^d_m\rangle$ and consider the corresponding operator $\mathcal {A}$. Then $(\langle x,g^d_m\rangle )_m=(\sum _n A_{m,n} c_n )_m = \mathcal {A}(c_n)\in \mathbf{s}$ (by Corollary 3.3), which implies that $x\in X_F$, and furthermore, for every $k\in {\mathbb {N}}$, one has $\Vert T_E (c_n)_n\Vert _{k}= \Vert x\Vert _{k}={\Vert |{(\langle x, g^d_m\rangle )_m}\Vert |}_{\Theta _k}$. For every $k\in {\mathbb {N}}$, there is a constant $R_k$ such that ${\Vert |{(d_n)}\Vert |}_{\Theta _k} \le R_k {\Vert |{(d_n)}\Vert |}_{\sup ,k+2}$ for every $(d_n)\in \mathbf{s}_{k+2}$. By Proposition 3.2, we conclude that
  $$\begin{aligned} \Vert T_E (c_n)_n\Vert _{k}\le & {} R_k {\Vert |{(\langle x, g^d_m\rangle )_m}\Vert |}_{\sup ,k+2}= R_k {\Vert |{\mathcal {A}(c_n)}\Vert |}_{\sup , k+2} \\\le & {} R_k K_{(s_0, k, C, C_k)} {\Vert |{(c_n)}\Vert |}_{\sup ,s_0 + k+4}. \end{aligned}$$
  Thus, the synthesis operator $T_E$ is well defined and continuous from $\mathbf{s}$ into $X_F$.
(iii) follows from (i) and (ii). $\square$

It is of interest to consider the case when $X_F$ is $\mathcal {S}$.

### Corollary 4.2

Let $(e_n)_{n=1}^\infty$ be a frame of $L^2({\mathbb {R}})$ with elements in $\mathcal {S}({\mathbb {R}})$. Assume that for every $k\in {\mathbb {N}}$ there are constants $C_k, \widetilde{C}_k$ such that

10 $$\begin{aligned} |\langle e_m, h_n\rangle | \le \left\{ \begin{array}{ll} C_k m^k n^{-k}, &{} n>m, \\ \widetilde{C}_k n^k m^{-k}, &{} n\le m. \end{array} \right. \end{aligned}$$

Then the analysis operator $U_E$ is continuous from $\mathcal {S}$ into $\mathbf{s}$, the synthesis operator $T_E$ is continuous from $\mathbf{s}$ into $\mathcal {S}$, and the frame operator $T_EU_E$ is continuous from $\mathcal {S}$ into $\mathcal {S}$.

Now, we consider sub-exponential weights.

### Proposition 4.3

Let $\beta \in (0,1)$ and let the assumptions of the first part of Lemma 2.2 hold with the weights $\mu _k(x) = e^{k|x|^\beta }$, $k\in {\mathbb {N}}_0$. Let $E=(e_n)_{n=1}^\infty$ be a sequence with elements from $X_F$ which is a frame for ${\mathcal {H}}$. Then the following statements hold.

(i) Assume that there exist constants $\gamma _0\in {\mathbb {N}}$, $C>0$ such that for every $k\in {\mathbb {N}}$ there exists $C_k>0$ such that
  11 $$\begin{aligned} |\langle e_m, g_n\rangle | \le \left\{ \begin{array}{ll} C \mathrm {e}^{\gamma _0n^{\beta }}, &{} n>m, \\ C_k \mathrm {e}^{k (n^{\beta }-m^{\beta })}, &{} n\le m, k\in {\mathbb {N}}. \end{array} \right. \end{aligned}$$
  Then the analysis operator $f\rightarrow U_Ef =(\langle f,e_m\rangle )_{m=1}^\infty$ is continuous from $X_F$ into ${\mathfrak {s}}^\beta$.
(ii) Assume that there exist constants ${\tilde{\gamma }}_0\in {\mathbb {N}}$, $\tilde{C}>0$ such that for every $k\in {\mathbb {N}}$ there exists ${\tilde{C}}_k>0$ such that
  12 $$\begin{aligned} |\langle e_m, g_n^d\rangle | \le \left\{ \begin{array}{ll} {\tilde{C}} \mathrm {e}^{{\tilde{\gamma }}_0m^{\beta }}, &{} m>n, \\ {\tilde{C}}_k \mathrm {e}^{k (m^\beta -n^\beta )}, &{} m\le n. \end{array} \right. \end{aligned}$$
  Then the synthesis operator $(c_n)_n\rightarrow T_E(c_n) =\sum c_n e_n$ is continuous from ${\mathfrak {s}}^\beta$ into $X_F$.
(iii) If (11) and (12) hold, then the frame operator $T_E U_E$ is continuous from $X_F$ into $X_F$.

### Proof

Under the given assumptions, $\Theta _F$ is the space ${\mathfrak {s}}^\beta$. The rest of the proof can be done in a similar way as the proof of Proposition 4.1, using Corollary 3.8 instead of Corollary 3.3. $\square$

If in the above proposition one chooses *G* to be the Hermite basis $(h_n)_{n=1}^\infty$ and $\beta =1/(2\alpha )$, $\alpha >1/2$, then $X_F=\Sigma ^\alpha$.

### Corollary 4.4

Let $\alpha >1/2$. Let $(e_n)_{n=1}^\infty$ be a sequence with elements from $\Sigma ^\alpha$ which is a frame for $L^2({\mathbb {R}})$ and such that for every $k\in {\mathbb {N}}$ there are constants $C_k, \widetilde{C}_k$ such that

$$\begin{aligned} |\langle e_m, h_n\rangle | \le C_k e^{-k|n^{1/(2\alpha )}-m^{1/(2\alpha )}|}, \ m,n,k\in {\mathbb {N}}. \end{aligned}$$

Then the analysis operator $U_E$ is continuous from $\Sigma ^\alpha$ into ${\mathfrak {s}}^{1/(2\alpha )}$, the synthesis operator $T_E$ is continuous from ${\mathfrak {s}}^{1/(2\alpha )}$ into $\Sigma ^\alpha$, and the frame operator $T_EU_E$ is continuous from $\Sigma ^\alpha$ into $\Sigma ^\alpha$.

## Boundedness and Banach Frames Derived from Sub-exponential Localization of Frames

In this section we extend statements from [23] for polynomially and exponentially localized frames to the case of sub-exponentially localized frames (Theorem 5.4 below). We will use the Jaffard’s theorem [27] given there for the sub-exponential and exponential case (see Theorem 5.2 below).

First recall the *Schur’s test*: *If* $(A_{m,n})_{m,n\in {\mathbb {N}}}$ *is an infinite matrix satisfying* $\sup _{m\in {\mathbb {N}}} \sum _{n\in {\mathbb {N}}} |A_{m,n}| \le K_1$ *and* $\sup _{n\in {\mathbb {N}}} \sum _{m\in {\mathbb {N}}} |A_{m,n}| \le K_2$, *then the corresponding matrix type operator* $\mathcal {A}$ *is well defined and bounded from* $\ell ^p$ *into* $\ell ^p$ *for* $1\le p\le \infty$ *and the operator norm* $\Vert \mathcal {A}\Vert _{\ell ^p\rightarrow \ell ^p}\le K_1^{1/p'} K_2^{1/p}$.

Let $\beta \in (0,1]$ and $\gamma \in (0,\infty )$. Define ${\mathcal {E}}_{\gamma ,\beta }$ to be the space of matrices $(A_{m,n})_{m,n\in {\mathbb {N}}}$ satisfying the following condition:

13 $$\begin{aligned} \exists C_\gamma \in (0,\infty ) \ \text{ so } \text{ that } \ |A_{m,n}|\le C_\gamma e^{-\gamma |m-n|^\beta }, m,n\in {\mathbb {N}}. \end{aligned}$$

By the Schur’s test, when $(A_{m,n})_{m,n\in {\mathbb {N}}}\in {\mathcal {E}}_{\gamma ,\beta }$, then the corresponding matrix type operator $\mathcal {A}$ is well defined and bounded from $\ell ^2$ into $\ell ^2$, and for the operator norm one has that $\Vert \mathcal {A}\Vert _{\ell ^2\rightarrow \ell ^2}\le 2C_\gamma P_{\gamma ,\beta }$, where $C_\gamma$ is the constant from (13) and $P_{\gamma ,\beta }$ denotes the sum of the convergent series $\sum _{j=0}^\infty e^{-\gamma j^{\beta }}$.

We also need the following statements, an extension of [23, Lemmas 2 and 3] with a sketch of a proof in the spirit of that paper. It should be noted that the statements can be traced back to [18] and [24, Secs. 2, 7].^1^

### Lemma 5.1

For every $\gamma \in (0,\infty )$ and $\beta \in (0,1]$, the following holds.

- There exists a positive number *C* so that $\sum _{k\in {\mathbb {N}}} e^{-\gamma |m-k|^\beta } e^{-\gamma |k-n|^\beta } \le C e^{-(\gamma /2) |m-n|^\beta }$ for every $m,n\in {\mathbb {N}}$.
- If the matrix $(A_{m,n})_{m,n\in {\mathbb {N}}}$ belongs to ${\mathcal {E}}_{\gamma ,\beta }$ and $\mu$ is a $\beta _\mu$-sub-exponential weight with $\beta _\mu <\beta$, then $\mathcal {A}$ maps boundedly $\ell ^p_\mu ({\mathbb {N}}_0)$ into $\ell ^p_\mu ({\mathbb {N}}_0)$ for every $p\in [1,\infty ]$.

### Proof

- can be proved following the idea of [23, Lemma 2].
- Let $\gamma _\mu$ come from the $\beta _\mu$-sub-exponential weight $\mu$, i.e., $\mu (t+x)\le C_\mu e^{\gamma _\mu |t|^{\beta _\mu }}\mu (x), \ t,x\in {\mathbb {R}}.$ This gives $\mu (x)=\mu (x-n+n) \le C_\mu e^{\gamma _\mu |x-n|^{\beta _\mu }}\mu (n),$ $x,n\in {\mathbb {N}}.$ Choose $k>\gamma \beta /(\gamma _\mu \beta _\mu )$. With this *k* and the assumption $\beta _\mu <\beta$, one obtains that there is a constant $C_1>0$ so that
  14 $$\begin{aligned} e^{-(\gamma /k) |x-n|^{\beta }}\mu (n)^{-1} \mu (x) \le C_1. \end{aligned}$$
  This follows from the fact that for the chosen *k* the function $f(u)= \gamma _\mu u^{\beta _\mu } -(\gamma /k) u^{\beta }, u\in [1,\infty ),$ is bounded.

The rest of the proof can be done by trivial adaptations using [23, Lemma 3], (extending *k* if it is necessary) and showing first that $\mathcal {A}$ maps boundedly $\ell ^p_\mu ({\mathbb {N}}_0)$ into $\ell ^p_\mu ({\mathbb {N}}_0)$ for $p=1$ and $p=\infty$ and then using interpolation. $\square$

### Theorem 5.2

(Jaffard) Given $\beta \in (0,1]$ and $\gamma \in (0,\infty )$, let $(A_{m,n})_{m,n\in {\mathbb {N}}}\in {\mathcal {E}}_{\gamma ,\beta }$ and let the corresponding matrix type operator $\mathcal {A}$ be invertible on $\ell ^2$. Then $\mathcal {A}^{-1}\in {\mathcal {E}}_{\gamma _1,\beta }$ for some $\gamma _1\in (0,\gamma )$.

In “Appendix” we will give a sketch of the Jaffard’s proof.

### Remark 5.3

The exponential localization type condition $|A_{m,n}|\le C_\gamma e^{-\gamma |m-n|}$, $m,n\in {\mathbb {N}}$, considered in [23], implies that

$$\begin{aligned} \forall \beta \in (0,1) \ \forall k\in {\mathbb {N}}\ \exists \widetilde{C_k} \text{ so } \text{ that } |A_{m,n}|\le \widetilde{C_k}e^{-k|m-n|^\beta }, m,n\in {\mathbb {N}}. \end{aligned}$$

Here we consider the more general case intrinsically related to $\beta \in (0,1)$.

### Theorem 5.4

Let $p\in [1,\infty )$ and *G* be a Riesz basis for ${\mathcal {H}}$, and let *E* be a frame for ${\mathcal {H}}$ which is $\beta$-sub-exponentially or exponentially localized (respectively, $(k+1+\varepsilon )$-localized for some $\varepsilon >0$) with respect to *G*. Let $\mu$ be $\beta _\mu$-sub-exponential weight and let $\beta _\mu <\beta$ in the case of $\beta$-sub-exponentially localized frame *E* (respectively, let $\mu$ be a *k*-moderate weight) with $\mu (n)\ge 1$ for every $n\in {\mathbb {N}}$ and $\ell ^p_\mu \subset \ell ^2$.

Then for every $p\in [1,\infty )$ the following statements hold.

(i) The analysis operator $U_E$ maps boundedly ${\mathcal {H}}_G^{\ell ^p_\mu }$ into $\ell ^p_\mu$.
(ii) The synthesis operator $T_E$ maps boundedly $\ell ^p_\mu$ into ${\mathcal {H}}_G^{\ell ^p_\mu }$.
(iii) The frame operator $S_E=T_E U_E$ is invertible on ${\mathcal {H}}_G^{\ell ^p_\mu }$ and the series in $S_Ef=\sum _{n=1}^\infty \langle f, e_n\rangle e_n$ converges unconditionally.
(iv) The canonical dual frame $(\widetilde{e_n})_{n=1}^\infty$ of $(e_n)_{n=1}^\infty$ has the same type of localization as $(e_n)_{n=1}^\infty$, i.e., it is $\beta$-sub-exponentially or exponentially localized (resp. $(k+1+\varepsilon )$-localized) with respect to *G*.
(v) The frame expansions $f=\sum _{n=1}^\infty \langle f,e_n\rangle \widetilde{e_n}= \sum _{n=1}^\infty \langle f,\widetilde{e_n}\rangle e_n$ hold with unconditional convergence in ${\mathcal {H}}_G^{\ell ^p_\mu }$.
(vi) There is norm equivalence between $\Vert f\Vert _{{\mathcal {H}}_G^{\ell ^p_\mu }}$, ${\Vert |{(\langle f,e_n\rangle )_{n=1}^\infty }\Vert |}_{\ell ^p_\mu }$, and ${\Vert |{(\langle f,\widetilde{e_n}\rangle )_{n=1}^\infty }\Vert |}_{\ell ^p_\mu }$.

### Proof

In the cases of polynomial and exponential localization, the assertions are given in [23, Prop. 8 and Prop. 10]. For the sub-exponential case, one can proceed in a similar way, but using Lemma 5.1 and Theorem 5.2. For the sake of completeness, we sketch a proof.

Let $\gamma >0$ and $C>0$ come from the sub-exponential localization of *E* with respect to *G*, i.e., $\max \{ |\langle e_m, g_n\rangle |, |\langle e_m, \widetilde{g_n}\rangle |\} \le C \mathrm {e}^{- \gamma |m-n|^\beta }, \ m,n\in {\mathbb {N}}.$ Consider the matrix $(A_{m,n})_{m,n\in {\mathbb {N}}}$ determined by $A_{m,n}=e^{-\gamma |m-n|^\beta }$ for $m,n\in {\mathbb {N}}$.

(i) Let $f\in {\mathcal {H}}_G^{\ell ^p_\mu }$ and thus $(|\langle f, \widetilde{g_n}\rangle |)_{n=1}^\infty \in {\ell ^p_\mu }$. By Lemma 5.1(b), we have that $\mathcal {A} (|\langle f, \widetilde{g_n}\rangle |)_{n=1}^\infty$ belongs to ${\ell ^p_\mu }$. Furthermore, for $m\in {\mathbb {N}}$ we have
  $$\begin{aligned} |\langle f, e_m\rangle |\le & {} \sum _{n=1}^\infty |\langle f, \widetilde{g_n}\rangle \langle g_n, e_m\rangle | \le C \sum _{n=1}^\infty A_{m,n} |\langle f, \widetilde{g_n}\rangle |. \end{aligned}$$
  Therefore, $(\langle f, e_m\rangle )_{m=1}^\infty$ also belongs to ${\ell ^p_\mu }$ and
  $$\begin{aligned} \Vert (\langle f, e_m\rangle )_{m=1}^\infty \Vert _{\ell ^p_\mu } \le C \Vert \mathcal {A} (|\langle f, \widetilde{g_n}\rangle |)_{n=1}^\infty \Vert _{\ell ^p_\mu } \le C\Vert \mathcal {A}\Vert \cdot \Vert f\Vert _{{\mathcal {H}}_G^{\ell ^p_\mu }}. \end{aligned}$$
(ii) Let $c=(c_n)_{n=1}^\infty \in \ell ^p_\mu (\subseteq \ell ^2)$. Then the series $\sum _{n=1}^\infty c_n e_n$ converges in ${\mathcal {H}}$ and let us denote its sum by *y*. Since $\mathcal {A}(|c_n|)_{n=1}^\infty \in \ell ^p_\mu$ by Lemma 5.1(b), and since $|\langle y, \widetilde{g}_m\rangle |\le C \sum _{n=1}^\infty |c_n | |\langle e_n, \widetilde{g}_m \rangle | \le C \sum _{n=1}^\infty A_{m,n} |c_n|$ for every $m\in {\mathbb {N}}$, it follows that $U_{\widetilde{G}} y \in \ell ^p_\mu$ and therefore the element $T_G U_{\widetilde{G}} y=y$ belongs to ${\mathcal {H}}_G^{\ell ^p_\mu }$. Hence, $T_E$ maps $\ell ^p_\mu$ into ${\mathcal {H}}_G^{\ell ^p_\mu }$ and furthermore, $\Vert T_E c\Vert _{{\mathcal {H}}_G^{\ell ^p_\mu }}=\Vert y\Vert _{{\mathcal {H}}_G^{\ell ^p_\mu }} = \Vert U_{\widetilde{G}} y\Vert _{\ell ^p_\mu } \le C \Vert \mathcal {A} (|c_n|)\Vert _{\ell ^p_\mu }$, which by Lemma 5.1 implies that $\Vert T_E c\Vert _{{\mathcal {H}}_G^{\ell ^p_\mu }} \le C \Vert \mathcal {A}\Vert \Vert c\Vert _{\ell ^p_\mu }$.
(iii) By (i) and (ii), $S_E$ maps boundedly ${\mathcal {H}}_G^{\ell ^p_\mu }$ into ${\mathcal {H}}_G^{\ell ^p_\mu }$. For the unconditional convergence, take any re-ordering $N_1$ of ${\mathbb {N}}$. Let $f\in {\mathcal {H}}_G^{\ell ^p_\mu }$. Consider $\sum _{n\in N_1}\langle f, e_n\rangle e_n$ and take $\varepsilon >0$. Since $(\langle f, e_n\rangle )_{n\in N_1}\in \ell ^p_\mu$, there is a finite set $N_2\subset N_1$ so that $\Vert (\langle f, e_n\rangle )_{n\in N_1\setminus N_2}\Vert _{\ell ^p_\mu } <\varepsilon$. Then, for every finite $N_3$ such that $N_3\supset N_2$, $N_3\subset N_1$, one has that $\Vert Sf-\sum _{n\in N_3}\langle f, e_n\rangle e_n\Vert \le \Vert T_E\Vert \Vert (\langle f, e_n\rangle )_{n\in N_1\setminus N_2}\Vert _{\ell ^p_\mu } < \Vert T_E\Vert \varepsilon .$ Therefore, $\sum _{n\in N_1}\langle f, e_n\rangle e_n$ converges to *Sf*.
  Finally, let us show the bijectivity of $S_E$ on ${\mathcal {H}}_G^{\ell ^p_\mu }$. Consider the operator $\mathcal {V}:= U_{\widetilde{G}} S_E T_G$ and observe that it is invertible on $\ell ^2$ and it maps boundedly $\ell ^p_\mu$ into $\ell ^p_\mu$. Let $(V_{m,n})_{m,n\in {\mathbb {N}}}$ be the corresponding matrix of $\mathcal {V}$. Since $|V_{m,n}| =|\langle S_E g_n, \widetilde{g_m}\rangle | \le \sum _i |\langle g_n, e_i\rangle |\cdot |\langle e_i, \widetilde{g_m}\rangle |$, by Lemma 5.1(a), there is a positive constant $C_1$ so that $|V_{m,n}| \le C_1 e^{-(\gamma /2)|m-n|^\beta }$. Now by Theorem 5.2 it follows that $\mathcal {V}^{-1}\in {\mathcal {E}}_{\gamma _1,\beta }$ for some $\gamma _1\in (0, \gamma /2)$. By Lemma 5.1, it follows that $\mathcal {V}^{-1}$ maps boundedly $\ell ^p_\mu$ into $\ell ^p_\mu$. Therefore, $\mathcal {V}$ is a bounded bijection of $\ell ^p_\mu$ onto $\ell ^p_\mu$. Now the representation $S_E=T_G \mathcal {V} U_{\widetilde{G}}$ implies that $S_E$ is a bounded bijection of ${\mathcal {H}}_G^{\ell ^p_\mu }$ onto ${\mathcal {H}}_G^{\ell ^p_\mu }$.
(iv) Use the operator $\mathcal {V}$, determined in (iii), and observe that for $m,n\in {\mathbb {N}}$ we have $\langle \widetilde{e_m}, g_n\rangle = \sum _{j=1}^\infty \langle e_m, g_j\rangle \overline{(V^{-1})_{jn}}$ and $\langle \widetilde{e_m}, \widetilde{g_n}\rangle = \sum _{j=1}^\infty \langle e_m, \widetilde{g_j}\rangle \overline{(V^{-1})_{jn}}$. Since $\mathcal {V}^{-1}\in {\mathcal {E}}_{\gamma _1,\beta }$ for some $\gamma _1\in (0, \gamma /2)$, one can apply Lemma 5.1(a) appropriately to conclude.
(v) follows from (iii) and for (vi) one can use the representations $f= S_E^{-1} S_E =S_{\widetilde{E}^{-1}} S_{\widetilde{E}}f$ and the already proved (i)-(iv). $\square$

## Expansions in Fréchet Spaces Via Localized Frames

Our goal is expansion of elements of a Fréchet space and its dual via localized frames and coefficients in a corresponding Fréchet sequence space. First we present in the next theorem general results related to frames localized with respect to a Riesz basis. In the next section, we will apply this theorem using frames localized with respect to the Hermite orthonormal basis in order to obtain frame expansions in the spaces $\mathcal {S}$ and $\Sigma ^\alpha$, $\alpha >1/2$, and their duals. To clarify notation, for an element $e\in {\mathcal {H}}$, its corresponding element in ${\mathcal {H}}^*$ by the Rieszs representation theorem will be denoted by the bold-style letter $\mathbf{e}$. Note that in the setting of Lemma 2.2, for an element *e* from $X_0$, one can conclude that $\mathbf{e}$ belongs to $X_0^*$; thus, for $e\in X_F(\subseteq X_0)$, we can consider $\mathbf{e}$ as an element of $X_F^*$.

### Theorem 6.1

Let *G* be a Riesz basis for ${\mathcal {H}}$, $k\in {\mathbb {N}}_0$, and $\mu _k$ be a $\beta _k$-sub-exponential (resp. *k*-moderate) weight so that (5) holds. Let the spaces $\Theta _k$ and $X_k$ be as in Lemma 2.2. Assume that $E=(e_n)_{n=1}^\infty$ is a sequence with elements in $X_F$ forming a frame for ${\mathcal {H}}$ which is $\beta$-sub-exponentially localized with $\beta >\beta _k$ for all $k\in {\mathbb {N}}_0$ or exponentially localized (respectively, *s*-localized for every $s\in {\mathbb {N}}$) with respect to *G*. Then, $\widetilde{e_n}\in X_F$, $n\in {\mathbb {N}}$, and the following statements hold:

(i) The analysis operator $U_E$ is *F*-bounded from $X_F$ into $\Theta _F$, the synthesis operator $T_E$ is *F*-bounded from $\Theta _F$ into $X_F$, and the frame operator $S_E$ is *F*-bounded and bijective from $X_F$ onto $X_F$ with unconditional convergence of the series in $S_Ef=\sum _{n=1}^\infty \langle f, e_n\rangle e_n$.
(ii) For every $f\in X_F$,
  15 $$\begin{aligned} f=\sum \limits _{n=1}^\infty \langle f, \widetilde{e_n} \rangle e_n = \sum _{n=1}^\infty \langle f, e_n\rangle \widetilde{e_n} (\hbox {with convergence in }X_F) \end{aligned}$$
  with $$(\langle f, \widetilde{e_n} \rangle )_{n=1}^\infty \in \Theta _F$$ and $$(\langle f, e_n \rangle )_{n=1}^\infty \in \Theta _F$$.
(iii) If $X_F$ and $\Theta _F$ have the following property with respect to $(g_n)_{n=1}^\infty$:
  $$\begin{aligned} {\mathcal {P}}_{(g_n)}:\hbox { For }f\in {\mathcal {H}},\hbox { one has }f\in X_F\hbox { if and only if }(\langle f, g_n \rangle )_{n=1}^\infty \in \Theta _F. \end{aligned}$$
  then $X_F$ and $\Theta _F$ also have the properties ${\mathcal {P}}_{(e_n)}$ and ${\mathcal {P}}_{(\widetilde{e_n})}$.
(iv) Both sequences $(\mathbf{e _n})_{n=1}^\infty$ and $(\widetilde{\mathbf{e _n}})_{n=1}^\infty$ form Fréchet frames for $X_F$ with respect to $\Theta _F$.
(v) For every $$g\in X_F^*$$,
  16 $$\begin{aligned} g = \sum \limits _{n=1}^\infty g(e_n) \, \widetilde{\mathbf{e _n}} =\sum _{n=1}^\infty g(\widetilde{e_n}) \, \mathbf{e _n} (\hbox {with convergence in }X_F^*) \end{aligned}$$
  with $$(g(e_n) )_{n=1}^\infty \in \Theta _F^*$$ and $$(g(\widetilde{e_n}) )_{n=1}^\infty \in \Theta _F^*$$.
(vi) If $(a_n)_{n=1}^\infty \in \Theta _F^*$, then $\sum _{n=1}^\infty a_n \mathbf{e} _n$ (resp. $\sum _{n=1}^\infty a_n \widetilde{\mathbf{e _n}}$) converges in $X_F^*$, i.e., the mapping $f\mapsto \sum _{n=1}^\infty \langle f, e_n\rangle a_n$ (resp. $f\mapsto \sum _{n=1}^\infty \langle f, \widetilde{e_n}\rangle a_n$) determines a continuous linear functional on $X_F$.

### Proof

(i) The properties for $U_E$, $T_E,$ and $S_E$ follow easily using Theorem 5.4(i)-(iii).
  Further, the bijectivity of $S_E$ on $X_F$ implies that $\widetilde{e_n}\in X_F$ for every $n\in {\mathbb {N}}$.
(ii) By Theorem 5.4(v), for every $$k\in {\mathbb {N}}$$ and every $$f\in X_k$$ we have that $$f=\sum _{n=1}^\infty \langle f, \widetilde{e_n} \rangle e_n = \sum _{n=1}^\infty \langle f, e_n\rangle \widetilde{e_n}$$ with convergence in $$ X_k$$. This implies that for every $$f\in X_F$$, one has that $$ f=\sum _{n=1}^\infty \langle f, \widetilde{e_n} \rangle e_n = \sum _{n=1}^\infty \langle f, e_n\rangle \widetilde{e_n}$$ with convergence in $$X_F$$.
  For every $k\in {\mathbb {N}}$ and every $f\in X_k$, by Theorem 5.4(i), we have that $(\langle f, e_n\rangle )_{n=1}^\infty \in \Theta _k$. Therefore, $(\langle f, e_n\rangle )_{n=1}^\infty \in \Theta _F$ for every $f\in X_F$. Furthermore, by Theorem 5.4(iv), $(\widetilde{e_n})_{n=1}^\infty$ has the same type of localization with respect to *G* as $(e_n)_{n=1}^\infty$. Thus, applying Theorem 5.4(i) with $(\widetilde{e_n})_{n=1}^\infty$ as a starting frame, we get that $(\langle f, \widetilde{e_n}\rangle )_{n=1}^\infty \in \Theta _F$ for $f\in X_F$.
(iii) If $f\in X_F$, it is already proved in (i) that $(\langle f, e_n \rangle )_{n=1}^\infty \in \Theta _F$ and $(\langle f, \widetilde{e_n} \rangle )_{n=1}^\infty \in \Theta _F$. To complete the proof of ${\mathcal {P}}_{(e_n)}$, assume that $f\in {\mathcal {H}}$ is such that $(\langle f, e_n \rangle )_{n=1}^\infty \in \Theta _F$. Consider
  $$\begin{aligned} (\langle f, g_n \rangle )_{n=1}^\infty = \left( \left\langle f, \sum _{j=1}^\infty \langle g_n,\widetilde{e_j}\rangle e_j \right\rangle \right) _{n=1}^\infty =\left( \sum _{j=1}^\infty \langle \widetilde{e_j}, g_n \rangle \langle f,e_j\rangle \right) _{n=1}^\infty . \end{aligned}$$
  Let $k\in {\mathbb {N}}$. Since $(\langle f, e_j \rangle )_{j=1}^\infty \in \ell ^2_{\mu _k}$ and by Theorem 5.4(iv), $(\widetilde{e_n})_{n=1}^\infty$ has the same type of localization with respect to *G* as $(e_n)_{n=1}^\infty$, it follows from Lemma 5.1(b) (for the case of sub-exponential localization) and from the way of the proof of [23, Lemma 3] (for the case of polynomial and exponential localization) that $(\sum _{j=1}^\infty \langle \widetilde{e_j}, g_n \rangle \langle f,e_j\rangle )_{n=1}^\infty \in \ell ^2_{\mu _k}$. Therefore, $(\langle f, g_n \rangle )_{n=1}^\infty \in \Theta _F$ and thus, by ${\mathcal {P}}_{(g_n)}$, it follows that $f\in X_F$. For completing the proof of ${\mathcal {P}}_{(\widetilde{e_n})}$, if $f\in {\mathcal {H}}$ is such that $(\langle f, \widetilde{e_n} \rangle )_{n=1}^\infty \in \Theta _F$, it follows in a similar way as above that $f\in X_F$.
(iv) By (i), $(\mathbf{e} _n(f))_{n=1}^\infty \in \Theta _F$ for $f\in X_F$, and by Theorem 5.4(vi), for $k\in {\mathbb {N}}$ and $f\in X_k$, the norms ${\Vert |{(\langle f,e_n\rangle )_{n=1}^\infty }\Vert |}_{\Theta _k}$ and $\Vert f\Vert _{X_k}$ are equivalent. Furthermore, it follows from Theorem 5.4 that the operator $V:=S_E^{-1} T_E\vert _{_{\Theta _F}}$ maps $\Theta _F$ into $X_F$ and it is *F*-bounded. Clearly, $V(\mathbf{e} _n(f))_{n=1}^\infty =f$, $f\in X_F$. Therefore, $(\mathbf{e} _n)_{n=1}^\infty$ is an *F*-frame for $X_F$ with respect to $\Theta _F$. In an analogue way, $(\widetilde{\mathbf{e _n}})_{n=1}^\infty$ is also an *F*-frame for $X_F$ with respect to $\Theta _F$.
(v) The representations in (i) can be re-written as $f=\sum _{n=1}^\infty \widetilde{\mathbf{e}_n} (f) e_n = \sum _{n=1}^\infty \mathbf{e}_n (f) \widetilde{e_n}$, $f\in X_F$, which implies validity of (16) for $g\in X_F^*$.
  For the rest of the proof, consider the *F*-bounded (and hence continuous) operator *V* from the proof of (iv) and observe that $\widetilde{e_n}=V\delta _n$, $n\in {\mathbb {N}}$. This implies that for $g\in X_F^*$ we have $(g(\widetilde{e_n}))_{n=1}^\infty =(gV (\delta _n))_{n=1}^\infty \in \Theta _F^\circledast$. With similar arguments, considering the operator $\widetilde{V}=S_{\widetilde{E}}^{-1} T_{\widetilde{E}}\vert _{_{\Theta _F}}$, it follows that $(g(e_n))_{n=1}^\infty \in \Theta _F^\circledast$.
(vi) Let $(a_n)_{n=1}^\infty \in \Theta _F^*$ and thus there is $k_0\in {\mathbb {N}}$ so that $(a_n)_{n=1}^\infty \in \Theta _{k_0}^*$, i.e., $C:=\sum _{n=1}^\infty |a_n|^2 |\mu _{k_0}(n)|^{-2}<\infty$. By Theorem 5.4(vi), there is a positive constant $B_{k_0}$ so that ${\Vert |{(\langle f, e_n\rangle )_{n=1}^\infty }\Vert |}_{\Theta _{k_0}} \le B_{k_0} \Vert f\Vert _{X_{k_0}}$ for every $f\in X_F$. Let $f\in X_F$. By (i), $(\langle f, e_n \rangle )_{n=1}^\infty \in \Theta _F$. Therefore, $\sum _{n=1}^\infty \langle f, e_n\rangle a_n$ converges and furthermore,
  $$\begin{aligned} |\sum _{n=1}^\infty \langle f, e_n\rangle a_n|^2\le & {} \left( \sum _{n=1}^\infty |\langle f, e_n\rangle |^2 |\mu _{k_0}(n)|^{2}\right) \left( \sum _{n=1}^\infty |a_n|^2 |\mu _{k_0}(n)|^{-2}\right) \\= & {} C {\Vert |{(\langle f, e_n\rangle )_{n=1}^\infty }\Vert |}_{\Theta _{k_0}} \le B_{k_0} C \Vert f\Vert _{X_{k_0}}, \end{aligned}$$
  which implies continuity of the linear mapping $f\mapsto \sum _{n=1}^\infty \langle f, e_n\rangle a_n$. In a similar way, it follows that $f\mapsto \sum _{n=1}^\infty \langle f, \widetilde{e_n}\rangle a_n$ determines a continuous linear functional on $X_F$. $\square$

### Remark 6.2

Note that in the setting of the above theorem, when *G* is an orthonormal basis of ${\mathcal {H}}$ or more generally, when *G* is a Riesz basis for ${\mathcal {H}}$ satisfying any of the following two conditions:

- $$(\mathcal {P}_1)$$: $$\forall s\in {\mathbb {N}}\ \, \exists C_s>0 \ : \ |\langle g_m, g_n\rangle |\le C_s(1+|m-n|)^{-s}, \ m,n\in {\mathbb {N}}$$,
- $$(\mathcal {P}_2)$$: $$\exists s>0 \ \, \exists C_s>0 \ : \ |\langle g_m, g_n\rangle | \le C_s \mathrm {e}^{-s |m-n|}, \ m,n\in {\mathbb {N}}, $$

then the property ${\mathcal {P}}_{(g_n)}$ is satisfied.

### Frame Expansions of Tempered Distributions and Ultradistributions

Here we apply Theorem 6.1 to obtain series expansions in the spaces $\mathcal {S}$ and $\Sigma ^\alpha$ ($\alpha >1/2$), and their dual spaces, via frames which are localized with respect to the Hermite basis.

#### Theorem 6.3

Assume that the sequence $(e_n)_{n=1}^\infty$ with elements from $\mathcal {S}({\mathbb {R}})$ (resp. in $\Sigma ^\alpha$) is a frame for $L^2({\mathbb {R}})$ which is polynomially (resp. sub-exponentially or exponentially) localized with respect to the Hermite basis $(h_n)_{n=1}^\infty$ with decay $\gamma$ for every $\gamma \in {\mathbb {N}}$. Let $(g_n)_{n=1}^\infty =(h_n)_{n=1}^\infty$. Then ${\mathcal {P}}_{(g_n)}$ and the conclusions in Theorem 6.1 hold with $X_F$ replaced by $\mathcal {S}$ (resp. $\Sigma ^\alpha$) and $\Theta _F$ replaced by $\mathbf{s}$ (resp. ${\mathfrak {s}}^{1/(2\alpha )}$).

#### Proof

For $k\in {\mathbb {N}}_0$, consider the *k*-moderate weight $\mu _k(x) = (1+|x|)^k$. The spaces $\Theta _k:=\ell ^2_{\mu _k}$, $k\in {\mathbb {N}}_0$, satisfy (1)-(2) and their projective limit $\Theta _F$ is the space $\mathbf{s}$. Consider the spaces $X_k:={\mathcal {H}}^{\Theta _k}_{(h_n)}$, $k\in {\mathbb {N}}_0$, which satisfy (1)-(2). As observed after Theorem 6.1, the property ${\mathcal {P}}_{(h_n)}$ is satisfied. Since for $f\in L^2({\mathbb {R}})$ one has that $f\in \mathcal {S}$ if and only if $(\langle f,h_n\rangle _{n=1}^\infty )\in \mathbf{s}$, it now follows that $X_F=\mathcal {S}$. Then the conclusions of Theorem 6.3 follow from Theorem 6.1.

The respective part of the theorem follows in a similar way. $\square$

As noticed in [35], having in mind the known expansions of tempered distributions $({\mathcal {S}}({\mathbb {R}}_+))'$ [16, 38] and Beurling ultradistributions $(G^\alpha _\alpha ({\mathbb {R}}_+))'$ [17, 25, 26], and their test spaces, by the use of the Laguerre orthonormal basis $l_n, n\in {\mathbb {N}},$ and validity of the corresponding properties ${\mathcal {P}}_{(l_n)}$, we can transfer the above results to the mentioned classes of distributions and ultradistributions over ${\mathbb {R}}_+.$

#### Remark 6.4

For Proposition 4.1 (resp. 4.3), it is of interest to consider cases when $X_F$ is the space $\mathcal {S}$ (resp. $\Sigma ^\alpha$). This was the case in Corollary 4.2 (resp. Corollary 4.4) taking *G* to be the Hermite basis. Based on Theorem 6.3, we can determine further cases. If *G* is a frame for $L^2({\mathbb {R}})$ with elements from $\mathcal {S}$ and polynomially localized with respect to $(h_n)_{n=1}^\infty$, then one also has $X_F=\mathcal {S}$. Indeed, under such assumptions on *G*, Theorem 6.3 implies that $f\in \mathcal {S}$ if and only if $(\langle f, \widetilde{g_n}\rangle )\in \mathbf{s}$, and by the construction of $X_F$, one has that $f\in X_F$ if and only if $(\langle f, \widetilde{g_n}\rangle )\in \mathbf{s}$.

Concerning Proposition 4.3, with $\alpha >1/2$ and $\beta =1/(2\alpha )$, we have a similar conclusion for $\Sigma ^\alpha$.

#### Example 6.5

As an illustration of Theorems 6.1 and 6.3, we give the next example. Let $r\in {\mathbb {N}}$ and for $i=1,2,\ldots ,r$, take $\varepsilon _i \ge 0$ and a sequence $(a_n^i)_{n=1}^\infty$ of complex numbers satisfying $|a_n^i|\le \varepsilon _i$ for $n\ge 2$, $\sum _{i=1}^r |a^i_1|\le 1$, and $\sum _{i=1}^r \varepsilon _i <1$. For $n\in {\mathbb {N}}$, consider $e_n:=h_n + \sum _{i=1}^r a_n^i h_{n+i}$. The sequence $(e_n)_{n=1}^\infty$ is a Riesz basis for $L^2({\mathbb {R}})$ and it is *s*-localized with respect to the Hermite orthonormal basis $(h_n)_{n=1}^\infty$ for every $s>0$, as well as exponentially localized with respect to $(h_n)_{n=1}^\infty$. In order to show that $(e_n)_{n=1}^\infty$ is a Riesz basis for $L^2({\mathbb {R}})$, we will represent $(e_n)_{n=1}^\infty$ as a sequence $(Uh_n)_{n=1}^\infty$ for some bounded bijective operator from $L^2({\mathbb {R}})$ onto $L^2({\mathbb {R}})$ using similar techniques as in [7, Example 1]. Define *U* by $Uh_n:=h_n + \sum _{i=1}^r a_n^i h_{n+i}$, $n\in {\mathbb {N}}$, and by linearity on the linear span of $h_n, n\in {\mathbb {N}}$. The obtained operator is bounded on the linear span, so extend it by continuity on $L^2({\mathbb {R}})$, leading to a bounded operator on $L^2({\mathbb {R}})$. It remains to show the bijectivity of *U*. Let $f\in L^2({\mathbb {R}})$. Then

$$\begin{aligned} Uf - \langle f,h_1\rangle h_1 =\sum _{n=2}^\infty \langle f,h_n\rangle h_n + \sum _{n=1}^\infty \sum _{i=1}^r a^i_n \langle f,h_n\rangle h_{n+i} \in \overline{span}\{h_n\}_{n=2}^\infty \end{aligned}$$

and thus, $\Vert Uf \Vert \ge |\langle f,h_1\rangle |$. Furthermore, for $f\in L^2({\mathbb {R}})$,

$$\begin{aligned} Uf=\sum _{n=1}^\infty \langle f,h_n\rangle Uh_n =f+ \sum _{n=1}^\infty \sum _{i=1}^r a^i_n \langle f,h_n\rangle h_{n+i}, \end{aligned}$$

leading to

$$\begin{aligned} \Vert Uf\Vert\le & {} \Vert f\Vert + \sum _{i=1}^r \Vert \sum _{n=1}^\infty a^i_n \langle f,h_n\rangle h_{n+i}\Vert =\Vert f\Vert +\sum _{i=1}^r \Vert (a^i_n \langle f,h_n\rangle )_{n=1}^\infty \Vert _{\ell ^2}\\\le & {} \Vert f\Vert +\sum _{i=1}^r \sqrt{|a^i_1|^2 \Vert f\Vert ^2 + \varepsilon _i^2 \Vert f\Vert ^2} \le \Vert f\Vert +\sum _{i=1}^r (|a^i_1| + \varepsilon _i) \Vert f\Vert )\\\le & {} 3\Vert f\Vert ,\\ \Vert Uf-f\Vert\le & {} \sum _{i=1}^r \sqrt{|a^i_1|^2 |\langle f,h_1\rangle |^2 + \sum _{n=2}^\infty |a^i_n|^2 |\langle f,h_n\rangle |^2 } \\\le & {} \Vert Uf\Vert + (\sum _{i=1}^r \varepsilon _i) \Vert f\Vert \\\le & {} \frac{3+\sum _{i=1}^r \varepsilon _i}{4}\Vert Uf\Vert +\frac{3+\sum _{i=1}^r \varepsilon _i}{4}\Vert f\Vert . \end{aligned}$$

Since $(3+\sum _{i=1}^r \varepsilon _i)/4<1$, it follows from [7, Lemma 1] that the bounded operator *U* is bijective on $L^2({\mathbb {R}})$ and thus $(e_n)_{n=1}^\infty$ is a Riesz basis for $L^2({\mathbb {R}})$.

Note that under the assumptions of the example, the classical way to obtain invertibility of *U* does not apply, because $\Vert U-Id\Vert$ is not necessarily smaller than 1.

#### Remark 6.6

As explained in [23], when dealing with Gabor frames and localization, the natural bases to be considered in this respect are the Wilson bases, but then Gabor frames are not localized with respect to a Wilson basis in the strict sense of the definition of polynomial and exponential localization. However, under appropriate conditions, the authors of [23] still obtain statements in the spirit of Theorem 5.4 which now leads to conclusions as in Theorem 6.1.

## Appendix

We add in Jaffard’s proof some comments in the end. We also give a simple known assertions for the class ${\mathcal {E}}_{\gamma ,\beta }$ defined on page 12.

### Lemma 7.1

Let $$\mathcal {A}\in {\mathcal {E}}_{\gamma ,\beta }$$.

(i) If *B* belongs to ${\mathcal {E}}_{\gamma ',\beta }$ for some $\gamma '\in (0,\gamma )$, then *AB* belongs to ${\mathcal {E}}_{\gamma ',\beta }$.
(ii) If *B* belongs to ${\mathcal {E}}_{\gamma ,\beta }$, then *AB* belongs to ${\mathcal {E}}_{\gamma ',\beta }$ for every $\gamma '\in (0,\gamma )$.

### Proof

(i) Under the assumptions, there exist positive constants $C_A$ and $C_B$ so that $\ |A_{m,n}|\le C_A e^{-\gamma |m-n|^\beta }$ and $\ |B_{m,n}|\le C_B e^{-\gamma ' |m-n|^\beta }$ for $m,n\in {\mathbb {N}}$. Further, using $|m-k|^{\beta }+|k-n|^\beta \ge |m-n|^\beta ,$ for every $m,n\in {\mathbb {N}}$, one has
  $$\begin{aligned} |(AB)_{m,n}| \le C_A C_B \sum _{k=1}^{\infty } e^{-\gamma '|m-k|^\beta }e^{-\gamma |k-n|^\beta } \le C_A C_B e^{-\gamma ' |m-n|^\beta } 2\sum _{j=0}^{\infty } e^{-(\gamma -\gamma ') j^\beta }. \end{aligned}$$
(ii) follows from (i). $\square$

Now we give some details for the Jaffard’s proof of Theorem 5.2, providing explicit estimates for the bounds.

As in [27], $$AA^*$$ belongs to $${\mathcal {E}}_{\gamma ',\beta }$$ for $$\gamma '\in (0,\gamma )$$ by Lemma 7.1, $$AA^*=\Vert AA^*\Vert (Id -R)$$ for some operator *R* with $$ ||R||=r<1$$, and $$(AA^*)^{-1}=\Vert AA^*\Vert ^{-1} \sum _{n=0}^\infty R^n$$. With the method from [27], one obtains

$$\begin{aligned} \sum _{k=1}^\infty |(R^k)_{m,n}|\le \sum _{k=1}^\infty \min \{r^k, (C_1)^k (2P)^{k-1}e^{\gamma '' |m-n|^\beta }\} \end{aligned}$$

where $\gamma ''\in (0,\gamma ')$, $C_1=1+\frac{C_{AA^*}}{\Vert AA^*\Vert }$ (with $C_{AA^*}$ satisfying $\ |(AA^*)_{m,n}|\le C_{AA^*} e^{-\gamma ' |m-n|^\beta }$ for every $m,n\in {\mathbb {N}}$), and $P=P_{\gamma '-\gamma '',\beta }= \sum _{j=0}^\infty e^{-(\gamma '-\gamma '')j^{\beta }}.$

For k=0, $(R^k)_{m,n}$ equals 1 when $m=n$ and 0 otherwise, so we have $|(R^0)_{m,n}|\le e^{-p |m-n|^\beta }$ for every *p*.

Fix $|m-n|$ and let $\varepsilon \in (0,1)$. With $K:=C_1 2P (>1)$, let

$$\begin{aligned} \Lambda _{|m-n|}:= & {} \{j\in {\mathbb {N}} \ : \ K^je^{-\gamma ''(1-\varepsilon )|m-n|^\beta }\le r^j\}\\= & {} \left\{ j\in {\mathbb {N}} \ : \ j\le \frac{\gamma ''(1-\varepsilon )|m-n|^\beta }{\ln (\frac{K}{r})} \right\} . \end{aligned}$$

Denote by $n_0$ the highest natural number such that $n_0\in \Lambda _{|m-n|}.$ Then

$$\begin{aligned} \sum _{k=1}^{n_0} |(R^k)_{m,n}|\le & {} \frac{1}{2P} \sum _{k=1}^{n_0} K^k e^{-\gamma ''(1-\varepsilon )|m-n|^\beta } e^{-\varepsilon \gamma ''|m-n|^\beta }\\\le & {} \frac{1}{2P} e^{-\varepsilon \gamma ''|m-n|^\beta } \sum _{k=1}^\infty r^k = \frac{r}{1-r} \frac{1}{2P} e^{-\varepsilon \gamma ''|m-n|^\beta }. \end{aligned}$$

For $n>n_0$ we have $r^n<r^{n_0}=e^{n_0\ln r}$ and hence,

$$\begin{aligned} \sum _{k=n_0+1}^{\infty } |(R^{k})_{m,n}| \le \sum _{k=n_0+1}^{\infty } r^k = r^{n_0+1}\sum _{k=0}^\infty r^k < \frac{1}{1-r} e^{- \gamma ''(1-\varepsilon ) \frac{\ln (1/r)}{\ln (K/r)} |m-n|^\beta }. \end{aligned}$$

Let

$$\begin{aligned} \gamma _1= \min \left\{ \frac{\ln 1/r}{\ln K/r}{\gamma ''(1-\varepsilon ), \varepsilon \gamma ''} \right\} . \end{aligned}$$

Therefore,

$$\begin{aligned} \sum _{{k}=0}^\infty |(R^{k})_{m,n}| \le e^{- \gamma _1 |m-n|^\beta } \left( 1 + \frac{r}{1-r}\cdot \frac{1}{2P} + \frac{1}{1-r}\right) . \end{aligned}$$

Now using the representation $A^{-1}=A^*(AA^*)^{-1}$ and Lemma 7.1, we can conclude that

$$\begin{aligned} |(A^{-1})_{m,n}|\le & {} C_A \Vert AA^*\Vert ^{-1} \left( 1 + \frac{r}{1-r}\cdot \frac{1}{2P} + \frac{1}{1-r}\right) 2P_{\gamma -\gamma _1,\beta } e^{-\gamma _1|m-n|^\beta }, \end{aligned}$$

where $C_A$ is a positive constant such that $|A_{m,n}|\le C_A e^{-\gamma |m-n|^\beta }$ for $m,n\in {\mathbb {N}}$.

## Footnotes

1 We are thankful to the referee for pointing us to Refs. [18, 24].
